# External validation of risk prediction scores in patients undergoing anatomic video-assisted thoracoscopic resection

**DOI:** 10.1007/s00464-022-09786-7

**Published:** 2022-12-07

**Authors:** Florian Ponholzer, Karol Chorazy, Caecilia Ng, Florian Kocher, Herbert Maier, Paolo Lucciarini, Dietmar Öfner, Florian Augustin

**Affiliations:** 1 Department of Visceral, Transplant and Thoracic Surgery, Center of Operative Medicine, Medical University of Innsbruck, Anichstrasse 35, 6020 Innsbruck, Austria; 2 Department of Internal Medicine V: Hematology and Oncology, Medical University Innsbruck, Innsbruck, Austria

**Keywords:** Lung cancer, Risk score, EuroLung, Morbidity, Mortality, VATS

## Abstract

**Background:**

EuroLung Risk scores were established to predict postoperative morbidity and mortality in patients undergoing anatomic lung resections. We aimed to perform an external validation of the EuroLung scores, which were calculated from data of the European Society of Thoracic Surgeons database, in our video-assisted thoracoscopic surgery cohort.

**Methods:**

All available EuroLung scores were calculated for 718 patients scheduled for anatomic video-assisted thoracoscopic surgery resections between 2009 and 2019. Morbidity and mortality according to the definitions of the EuroLung scores were analyzed in a prospectively maintained database.

**Results:**

Overall observed complication rate was 10.45%. Scores showed weak individual correlation (*η* = 0.155–0.174). The EuroLung1 app score showed the biggest area under the receiver operative characteristic (ROC) curve with 0.660. Binary logistic regression analysis showed that predicted postoperative forced expiratory volume in 1 s was associated with increased complications in both EuroLung1 and parsimonious EuroLung1 scores. Thirty-day mortality was 0.7% (predicted 1.10–1.40%) and was associated with predicted postoperative forced expiratory volume in 1 s for both EuroLung2 and parsimonious EuroLung2 scores. The EuroLung2 (2016) showed the biggest area under the ROC curve with 0.673. Only a very weak eta correlation between predicted and observed mortality was found for both aggregate EuroLung2, EuroLung2 (2016), EuroLung2 (2019), and parsimonious EuroLung2 (2016) (*η* = 0.025/0.015/0.011/0.009).

**Conclusion:**

EuroLung scores help to estimate postoperative morbidity. However, even with the highest aggregate EuroLung scores possible only 50% suffer from postoperative morbidity. Although calibration of the scores was acceptable, discrimination between predicted and observed events was poor. Therefore, individual correlation between predicted and observed events is weak. Therefore, EuroLung scores may be best used to compare institutional quality of care to the European Society of Thoracic Surgeons database but should not be used to preclude patients from surgical treatment.

**Supplementary Information:**

The online version contains supplementary material available at 10.1007/s00464-022-09786-7.

In the coming years the rate of surgically resectable early stage lung cancers will be on the rise, as lung cancer screening routines are established [1, 2]. As a result interdisciplinary teams will face the challenge to choose the right patient pathways and treatment modalities to achieve the best outcome for each patient. Although the gold standard for the treatment of UICC stage I cancer remains primary surgical resection, comorbidities might propose non-surgical treatment options [3].

Treatment planning in resectable lung cancer usually relies on an algorithm evaluating the fitness for surgery and is mainly focused on cardiac and pulmonary function testing. However, there are other medical conditions increasing the risk of postoperative complications and surgical mortality that are currently not routinely considered. Thoracic surgeons are trying to establish risk scores which may provide some decision guidance when interdisciplinary tumor boards have to weigh a primary surgical therapy approach against other treatment modalities, such as stereotactic body radiotherapy or radiofrequency ablation. The ESTS EuroLung scores, based on the ESTS database, represent such a tool for the risk calculation of 30-day postoperative morbidity and 30-day postoperative mortality based on clinical values [4–6]. The ESTS EuroLung scores consist of two groups of scores: EuroLung1 scores for morbidity and EuroLung2 scores for mortality, with parsimonious variants for both to simplify calculation. Aggregate EuroLung scores have been established for grouping patients in different classes of risk for morbidity and mortality. Moreover, the two scores have been updated in 2019 from its original version published in 2016. EuroLung scores have also been published in form of a freely available app for calculating EuroLung scores on a smartphone [4, 5]. The EuroLung scores have been applied to European, Canadian, and Japanese cohorts with inconclusive results [7, 8]. This study compares the EuroLung scores from 2016, 2019 and the app and is the first to test them in a pure video-assisted thoracoscopic surgery (VATS) cohort, while the scores originally were calculated using data from both, open and minimally invasive approaches.

## Materials and methods

### Patient selection

All patients scheduled for an anatomic VATS resection for primary lung cancer at our department from 02/2009 to 07/2019 were retrospectively analyzed. Due to our ethical directive patients under the age of 18 (*n* = 4) were excluded. To preemptively avoid possible confounders patients over the age of 80 (*n* = 20) were excluded, because this patient cohort represents a very preselected group with above-average performance status at our department (no mortality and 4.2% morbidity). No other exclusion criteria were used. 718 patients remained for further analysis. Permission for retrospective data analysis of our VATS cohort was granted by the local ethics committee (Registration Number: AN5163,327/4.17,382/5.2).

### Data administration

Data was collected in a prospectively maintained database. Collected data included sex, age at operation, coronary artery disease (CAD), chronic kidney disease (CKD), cerebrovascular disease (CVD), body mass index (BMI), type of resection (e.g., extended resection), predicted postoperative forced expiratory volume in one second (ppoFEV1), postoperative morbidity, death.

Postoperative morbidity was defined according to the EuroLung score and included, but was not limited to: respiratory failure, prolonged mechanical ventilation (> 24 h), acute heart failure, reintubation, pneumonia, atelectasis requiring a bronchoscopy, pulmonary edema, prolonged air leak, embolism, acute respiratory distress syndrome (ARDS), stroke, transient ischemic attack (TIA), acute kidney failure, arrhythmia requiring treatment, myocardial infarction. In accordance with the definition of the EuroLung scores postoperative morbidity and mortality was only included if it occurred during the first 30 days after surgery.

Pneumonia definition in our database matches the definition of Fernandez et al. [9].

### EuroLung scores

EuroLung1 and EuroLung2 score from 2016 and 2019, seen in Table 1, have been calculated using data from 47,960, respectively, 82,383, anatomic lung resections documented in the ESTS database (07/2007–08/2015 and 01/2007–12/2018) [4, 5].

**Table 1:** Evolution of EuroLung scores over time (EuroLung App: formula based on Brunelli et al. [4] and Pompili et al. [7])

*Variables*	*EuroLung scores*									
	EuroLung1(2016)	EuroLung1(2019)	Parsimonious EuroLung1(2016)	Parsimonious EuroLung1(2019)	EuroLung1 aggregate score(2016)	EuroLung2(2016)	EuroLung2(2019)	Parsimonious EuroLung2(2019)	EuroLung2 aggregate score(2016)	EuroLung2 aggregate score(2019)
Constant	−2.465	−2.821	−3.079	−2.852		−5.82	−6.36	−6.35		
Age	0.026	0.02	0.025	0.021		0.044	0.046	0.047		
Age > 65					3				2	
Age > 70										1
Male sex	0.497	0.455	0.477	0.472	3	0.903	0.866	0.889	3	2.5
ppoFEV_1_%	−0.015	−0.015	−0.015	−0.015		−0.009	−0.009	−0.010		
ppoFEV_1_ < 70%					3				1	1
Conversion/Thoracotomy	0.497	0.66	0.639	0.662	3	0.894	0.889	0.892	3	2.5
Extended resection	0.514	0.332	0.321	0.324	3	0.3	0.333		1	
Pneumectomy						0.929	0.968	0.983	3	3
CAD according to ESTS	0.231	0.206			2	0.264	0.184		1	
CVD according to ESTS	0.37	0.205			2	0.582	0.363		2	
CKD	0.152	0.214			1					
BMI						−0.064	−0.055	−0.055		
BMI < 18.5									3	2.5

*ppoFEV1* predicted postoperative forced expiratory volume in 1 s; *CAD* coronary artery disease; *ESTS* European society of thoracic surgeons; *CVD* cerebrovascular disease; *CKD* chronic kidney disease; *BMI* body mass index

### Statistical analysis

Statistical analysis was performed using IBM SPSS Statistics 26 (IBM Corporation, Armonk, NY, USA) and included the methods recommended by Altman et al. for external validation of prognostic tests [10].

Pearson’s chi-squared test or Fisher’s exact test were used for identifying relationships between categorical variables. One-way analysis of variance was used for comparing means between various numerical variables. In all EuroLung scores binary logistic regression was used to test their computational variables for significance between our patient groups with and without EuroLung morbidity/mortality. This is to examine whether the variables of our cohort differ from the study by Brunelli et al. [4]. Hosmer–Lemeshow-Test was used for testing for goodness of fit for the logistic regression scores. Area under the receiver operating characteristic curve (AUROC) was calculated to compare predictivity of the scores. For comparison of AUROC between the available scores DeLong test was used. For analysis of the relationship between nominal and metric variables eta correlation was used to calculate the correlation between a score and the observed morbidity/mortality via-cross tabulation. Calibration was assessed by using calibration-in-the-large and calibration slope. The study was performed in accordance to the TRIPOD statement for Prediction Model Validation [11].

Results were expressed as means. Statistical significance was assumed for a *p*-value < 0.05.

## Results

A total of 718 patients were analyzed. Overall patient characteristics and respective morbidity and mortality characteristics are shown in Supplementary Table 1, 2, 3, and 4.

Every patient in the cohort was scheduled for a primary anatomic VATS resection for primary lung cancer (100%). Our observed 30-day morbidity was 10.45% and observed 30-day mortality was 0.70%.

### Morbidity

In our cohort 75 out of 718 patients (10.45%) suffered from postoperative morbidity, as defined by Brunelli et al., in the first 30 days after surgery and this rate was lower than the calculated EuroLung scores (Table 2) [4, 5]. The relationship between 30-day morbidity and demographic data, risk scores, and perioperative morbidities are shown in Table 2.

**Table 2:** Relationship between 30-day morbidity and demographic data, risk scores, and perioperative morbidities

Variables	Results		Observed EuroLung-morbidity				*p*
			Yes (*n* = 75, 10.45%)		No (*n* = 643, 89.55%)		
Age	63.64	(10.05)	65.61	(9.71)	63.41	(10.07)	.073
Women	328	(45.68%)	23	(30.67%)	305	(47.43%)	.007
Men	390	(54.43%)	52	(69.33%)	338	(52.57%)	
BMI	25.34	(4.53)	25.30	(5.22)	25.34	(4.44)	.946
FEV_1_%	80.80	(17.08)	76.28	(17.32)	81.33	(16.98)	.015
ppoFEV_1_%	62.92	(15.06)	58.61	(14.20)	63.43	(15.09)	.009
CKD	42	(5.85%)	9	(12.00%)	33	(5.13%)	.032
CAD according to ESTS	62	(8.64%)	9	(12.00%)	53	(8.24%)	.277
CVD according to ESTS	31	(4.32%)	6	(8.00%)	25	(3.89%)	.124
Hemoglobin (preoperative)	13.78	(3.21)	14.65	(9.09)	13.68	(1.41)	.014
Creatinine (preoperative)	.93	(.41)	.99	(.37)	.93	(.42)	.255
Diabetes	90	(12.53%)	15	(20.00%)	75	(11.66%)	.044
Hypertension	292	(40.67%)	37	(49.33%)	255	(39.66%)	.136
Neoadjuvant therapy	73	(10.17%)	9	(12.00%)	64	(9.95%)	.686
Right lung	437	(60.86%)	49	(65.33%)	388	(60.34%)	.454
Left lung	281	(39.14%)	26	(34.67%)	255	(39.66%)	
Peripheral tumor	539	(75.07%)	55	(73.33%)	484	(75.74%)	.671
Central tumor	175	(24.37%)	20	(27.78%)	155	(24.26%)	
VATS lobectomy	618	(86.07%)	64	(85.53%)	553	(86.00%)	1.000
VATS sleeve resection	15	(2.09%)	2	(2.67%)	13	(2.02%)	.664
VATS bilobectomy	29	(4.04%)	5	(6.67%)	24	(3.73%)	.214
VATS segmentectomy	34	(4.74%)	4	(5.33%)	30	(4.67%)	.773
VATS pneumectomy	20	(2.79%)	0	(0%)	20	(3.11%)	.253
VATS completion pneumectomy	3	(.42%)	0	(0%)	3	(.47%)	1.000
Thoracotomy	0	(0%)	0	(0%)	0	(0%)	
Extended resection	10	(1.39%)	3	(4.00%)	7	(1.09%)	.077
Conversion	41	(5.71%)	8	(10.67%)	33	(5.13%)	.063
Removed segments	4.18	(1.49)	4.31	(1.54)	4.17	(1.48)	.446
EuroLung1 (2016)	20.85	(8.96)	25.27	(10.85)	20.33	(8.58)	< 0.001
EuroLung1 (2019)	11.16	(5.47)	13.86	(7.30)	10.85	(5.13)	< 0.001
Parsimonious EuroLung1 (2016)	11.45	(5.41)	13.91	(6.53)	11.16	(5.19)	< 0.001
Parsimonious EuroLung1 (2019)	11.11	(5.17)	13.45	(6.31)	10.84	(4.95)	< 0.001
EuroLung1 according to the EuroLung App	13.26	(8.10)	17.36	(10.54)	12.78	(7.63)	< 0.001
EuroLung1 aggregate score	5.68	(3.24)	6.84	(3.52)	5.54	(3.19)	.001
EuroLung2 (2016)	1.40	(1.54)	1.81	(1.49)	1.35	(1.54)	.016
EuroLung2 (2019)	1.11	(1.25)	1.42	(1.16)	1.08	(1.25)	.025
Parsimonious EuroLung2	1.10	(1.27)	1.36	(1.04)	1.07	(1.29)	.064
EuroLung2 according to the EuroLung App	1.29	(3.00)	1.39	(1.10)	1.27	(3.14)	.741
EuroLung2 aggregate score (2016)	3.86	(2.48)	4.73	(2.52)	3.76	(2.46)	.001
EuroLung2 aggregate score (2019)	2.66	(1.85)	3.31	(1.75)	2.58	(1.85)	.001
Observed 30-day mortality	5	(.70%)	4	(5.33%)	1	(.16%)	< 0.001

Results are shown as mean (standard deviation unless otherwise defined). *BMI* body mass index; *FEV1* forced expiratory volume in 1 s; *ppoFEV1* predicted postoperative forced expiratory volume in 1 s; *CKD* chronic kidney disease; *CAD* coronary artery disease; *ESTS* European society of thoracic surgeons; *CVD* cerebrovascular disease; *VATS* video-assisted thoracoscopic surgery

Using the various EuroLung scores, the calculated morbidity ranged from 11.11 to 20.85%. The parsimonious EuroLung1 (2019) showed the most accurate prediction with 11.11% (95%CI, 10.76–11.56%) in comparison to the cohorts observed morbidity rate of 10.45%. Patients with a postoperative morbidity showed significantly higher EuroLung scores in all available morbidity scores than patients without (EuroLung1 (2016): *p* =  < 0.001; EuroLung1 (2019): *p* =  < 0.001; parsimonious EuroLung1 (2016): *p* =  < 0.001; parsimonious EuroLung1 (2019): *p* =  < 0.001; EuroLung1 App: *p* =  < 0.001). Patients with morbidity also showed a significantly higher EuroLung1 aggregate score (6.84 (95%CI,6.03–7.65), vs 5.54 (95%CI,5.30–5.79); *p* = 0.001).

**Fig. 1:**
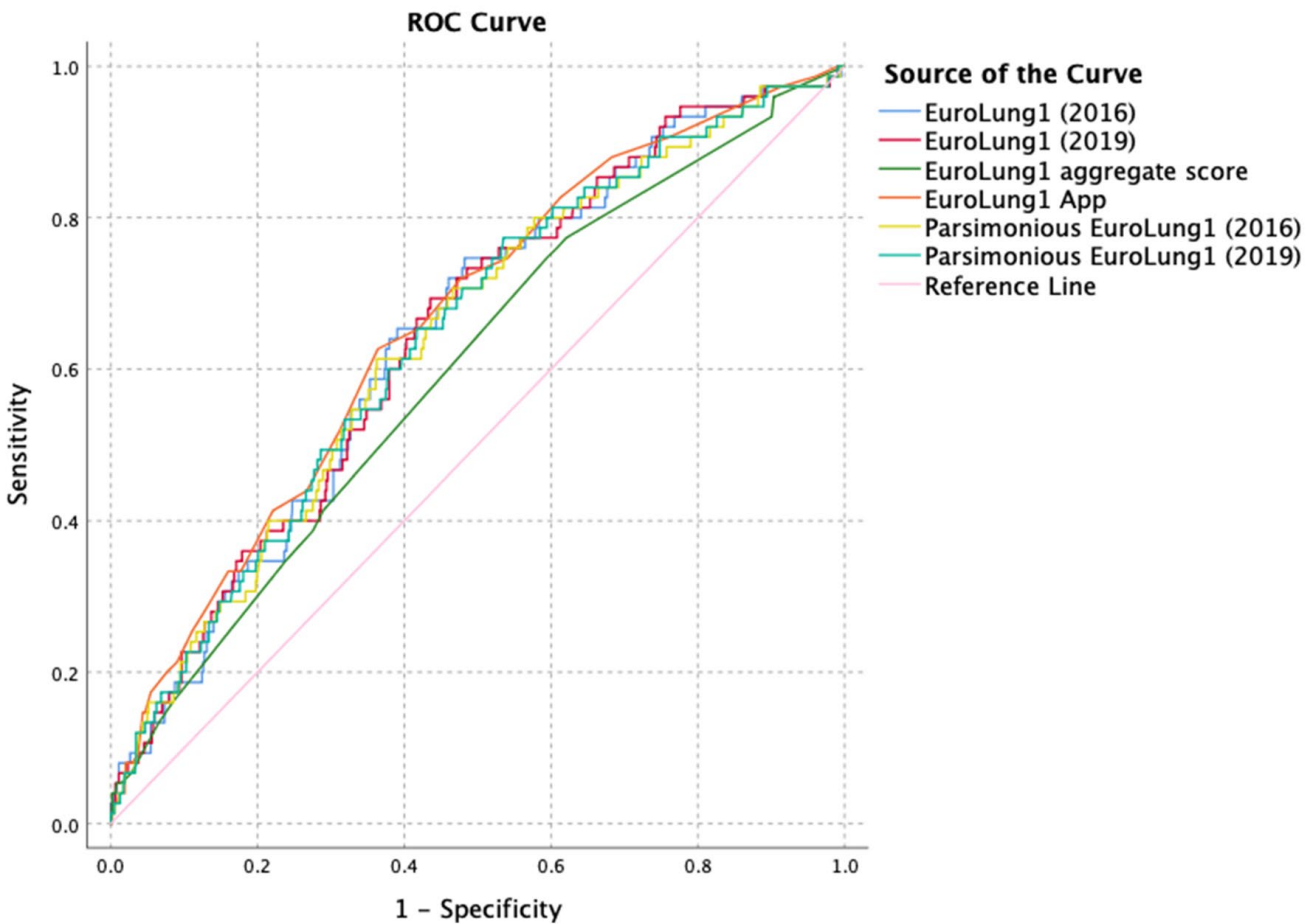
ROC-Curves of EuroLung1 scores

All EuroLung scores only showed a weak individual correlation (EuroLung1 (2016): *η* = 0.155; EuroLung1 (2019): *η* = 0.168; parsimonious EuroLung1 (2016): *η* = 0.156; parsimonious EuroLung1 (2019): *η* = 0.174; EuroLung1 App: *η* = 0.173; EuroLung1 aggregate score: *η* = 0.122). In accordance with these results the AUROC was 0.660 for the EuroLung1 App, 0.646 for the EuroLung1 (2019), 0.645 for the EuroLung1 (2016), 0.642 for both parsimonious EuroLung1 scores (2016 & 2019), 0.599 for the EuroLung1 aggregate score and did not proof high discrimination. The parsimonious Eurolung1 (2019), which showed the most accurate prediction and the highest *η*-value, had a statistically different AUROC than the EuroLung1 aggregate score (*p* = 0.010) and the EuroLung1 App (*p* = 0.032). The rest of the EuroLung scores showed no statistically different AUROC. The EuroLung1 App showed a significantly better discrimination than the EuroLung1 aggregate score (*p* = < 0.001) and both parsimonious EuroLung1 scores (*p* = 0.032/0.032), but not for the EuroLung1 (2016) (*p* = 0.220) and EuroLung1 (2019) (*p* = 0.217). Respective ROC curves are shown in Fig. 1.

The Hosmer–Lemeshow test for goodness of fit was not significant for all morbidity scores and therefore valid [EuroLung1 (2016 & 2019): *p* = 0.958; parsimonious EuroLung1 (2016 & 2019): *p* = 0.996; EuroLung1 aggregate score: *p* = 0.919].

**Fig. 2:**
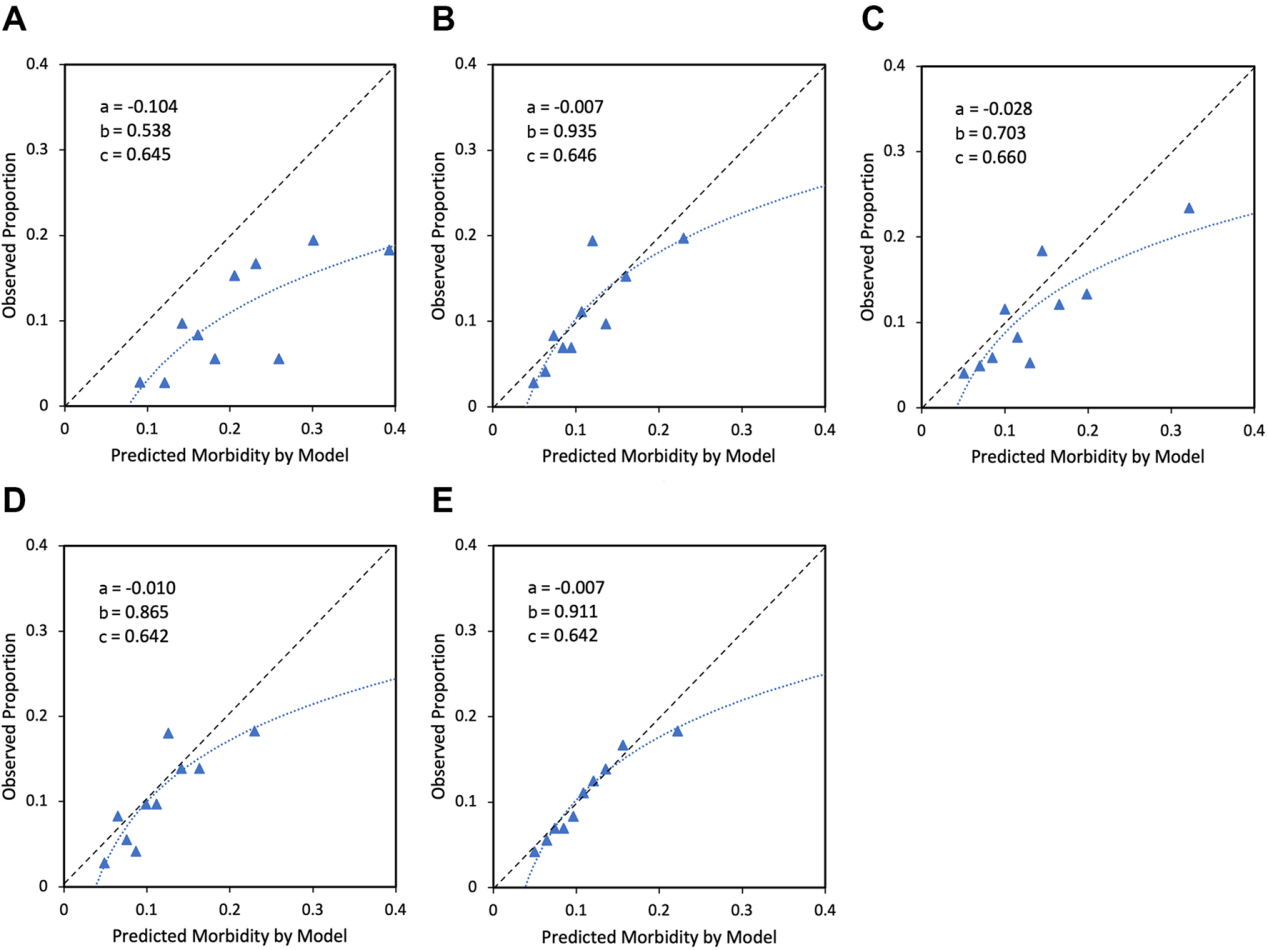
Calibration plots of **A** EuroLung1 (2016), **B** EuroLung1 (2019), **C** EuroLung1 App, **D** parsimonious EuroLung1 (2016) and **E** parsimonious EuroLung1 (2019). (a) Calibration-in-the-large calculated as the logistic regression model intercept given that the calibration slope equals 1; (b) calibration slope in a logistic regression model with the linear predictor as the sole predictor; (c) c-statistic indicating discriminative ability. Triangles represent deciles of subjects grouped by similar predicted risk

Calibration-in-the-large showed a graphical trend toward systematically too high predictions, while at the same time showing too extreme risk estimations in the calibration slope, as visualized in Fig. 2. EuroLung1 (2019) and parsimonious EuroLung1 (2019) showed the best calibration-in-the-large with an intercept close to 0 (*a* = −0.007/−0.007). Moreover, they also showed the tightest estimation spread with their respective calibration slopes being the closest to 1 (*b* = 0.935/0.911).

For further investigation of the impact of risk factors for morbidity a binary logistic regression analysis was performed for each risk score. For the EuroLung1 and parsimonious EuroLung1 (2016 & 2019) lower ppoFEV1% was associated with a higher risk for postoperative complications (EuroLung1 (2016 & 2019): *p* = 0.041, parsimonious EuroLung1 (2016 & 2019): *p* = 0.042). Male gender showed to be a significant risk factor for the aggregate EuroLung1 score (*p* = 0.025).

**Fig. 3:**
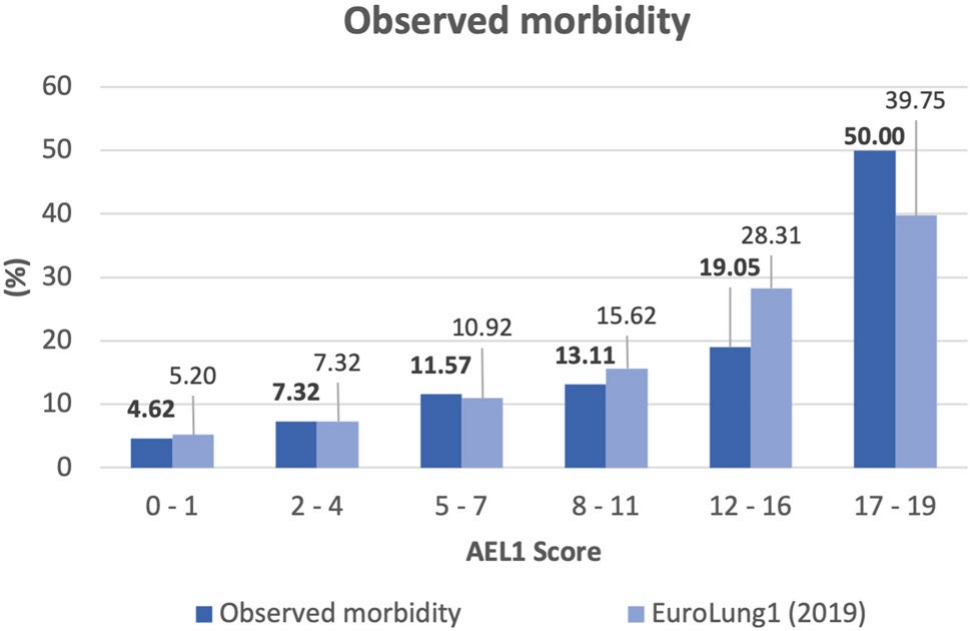
Relationship between EuroLung1 aggregate score and our morbidity rates. (*AEL1 Score* Aggregate EuroLung1 Score)

The relationship between the EuroLung1 aggregate score and our observed morbidity rate is shown in Fig. 3.

A subgroup analysis did not show a difference in observed morbidity for patients with neoadjuvant therapy (12.3 vs. 10.2% in patients without neoadjuvant therapy, *p* = 0.547).

### Mortality

Postoperative 30-day mortality in our cohort was observed in five patients (0.7%) and was lower than predicted in any EuroLung score. The closest result was estimated by the parsimonious EuroLung 2 with 1.10% (95%CI, 1.01–1.19%), followed by EuroLung2 (2019) with 1.11% (95%CI, 1.03–1.21%), EuroLung2 App with 1.29% (95%CI, 1.07–1.51%), and the EuroLung2 (2016) with 1.40% (95%CI, 1.29–1.51%) in comparison to the cohorts observed mortality rate of 0.7%. The relationship between 30-day mortality and demographic data, risk scores, and perioperative morbidities are shown in Table 3.

**Table 3:** Relationship between 30-day mortality and demographic data, risk scores, and perioperative morbidities

Variables	Results		Observed EuroLung-mortality				*p*
			Yes (*n* = 5, 0.70%)		No (*n* = 713, 99.30%)		
Age	63.64	(10.05)	65.40	(9.90)	63.63	(10.05)	.865
Women	328	(45.68%)	2	(40.00%)	328	(46.00%)	1.000
Men	390	(54.43%)	3	(60.00%)	390	(54.70%)	
BMI	25.34	(4.53)	22.13	(2.91)	25.36	(4.53)	.111
FEV_1_%	80.80	(17.08)	60.32	(6.65)	80.94	(17.04)	.007
ppoFEV_1_%	62.92	(15.06)	47.89	(8.01)	63.03	(15.05)	.025
CKD	42	(5.85%)	2	(40.00%)	40	(5.61%)	.030
CAD according to ESTS	62	(8.64%)	2	(40.00%)	60	(8.42%)	.062
CVD according to ESTS	31	(4.32%)	0	(0%)	31	(4.35%)	1.000
Hemoglobin (preoperative)	13.78	(3.21)	12.55	(1.13)	13.79	(3.22)	.443
Creatinine (preoperative)	.93	(.41)	1.56	(.82)	.93	(.41)	.001
Diabetes	90	(12.53%)	1	(16.67%)	89	(12.48%)	.489
Hypertension	292	(40.67%)	4	(80.00%)	288	(40.39%)	.164
Neoadjuvant therapy	73	(10.17%)	0	(0%)	73	(10.24%)	1.000
Right lung	437	(60.86%)	5	(100.00%)	432	(60.59%)	.163
Left lung	281	(39.14%)	0	(0%)	281	(39.41%)	
Peripheral tumor	539	(75.07%)	3	(60.00%)	536	(76.02%)	.601
Central tumor	175	(24.37%)	2	(40.00%)	173	(24.40%)	
VATS lobectomy	618	(86.07%)	5	(100.00%)	612	(85.83%)	1.000
VATS sleeve resection	15	(2.09%)	0	(0%)	15	(2.10%)	1.000
VATS bilobectomy	29	(4.04%)	0	(0%)	29	(4.07%)	1.000
VATS segmentectomy	34	(4.74%)	0	(0%)	34	(4.77%)	1.000
VATS pneumectomy	20	(2.79%)	0	(0%)	20	(2.81%)	1.000
VATS completion pneumectomy	3	(.42%)	0	(0%)	3	(.42%)	1.000
Thoracotomy	0	(0%)	0	(0%)	0	(0%)	
Extended resection	10	(1.39%)	0	(0%)	10	(1.40%)	1.000
Conversion	41	(5.71%)	0	(0%)	41	(5.75%)	1.000
Removed segments	4.18	(1.49)	3.60	(.89)	4.19	(1.49)	.381
EuroLung1 (2016)	20.85	(8.96)	26.27	(7.01)	20.81	(8.97)	.175
EuroLung1 (2019)	11.16	(5.47)	14.34	(3.51)	11.14	(5.48)	.192
Parsimonious EuroLung1 (2016)	11.45	(5.41)	13.51	(4.40)	11.43	(5.42)	.393
Parsimonious EuroLung1 (2019)	11.11	(5.17)	13.09	(4.17)	11.10	(5.17)	.393
EuroLung1 according to the EuroLung App	13.26	(8.10)	15.20	(5.63)	13.25	(8.11)	.591
EuroLung1 aggregate score	5.68	(3.24)	7.20	(2.17)	5.67	(3.25)	.293
EuroLung2 (2016)	1.40	(1.54)	1.67	(.77)	1.40	(1.54)	.695
EuroLung2 (2019)	1.11	(1.25)	1.28	(.57)	1.12	(1.25)	.769
Parsimonious EuroLung2	1.10	(1.27)	1.24	(.56)	1.10	(1.27)	.811
EuroLung2 according to the EuroLung App	1.29	(3.00)	1.26	(.57)	1.29	(3.01)	.983
EuroLung2 aggregate score (2016)	3.86	(2.48)	4.60	(.89)	3.86	(2.48)	.505
EuroLung2 aggregate score (2019)	2.66	(1.85)	3.20	(.67)	2.65	(1.86)	.510
Observed 30-day morbidity	75	(10.45%)	4	(80.00%)	71	(9.96%)	.001

Results are shown as mean (standard deviation unless otherwise defined). *BMI* body mass index; *FEV1* forced expiratory volume in 1 s; *ppoFEV1* predicted postoperative forced expiratory volume in 1 s; *CKD* chronic kidney disease; *CAD* coronary artery disease; *ESTS* European society of thoracic surgeons; *CVD* cerebrovascular disease; *VATS* video-assisted thoracoscopic surgery

Patients with observed mortality did not show significantly higher EuroLung scores (EuroLung2 (2016): *p* = 0.695; EuroLung2 (2019): *p* = 0.769; parsimonious EuroLung2: *p* = 0.811; EuroLung2 App: *p* = 0.983). Also, EuroLung2 aggregate scores (2016 & 2019) did not differ between groups (*p* = 0.505, *p* = 0.510).

**Fig. 4:**
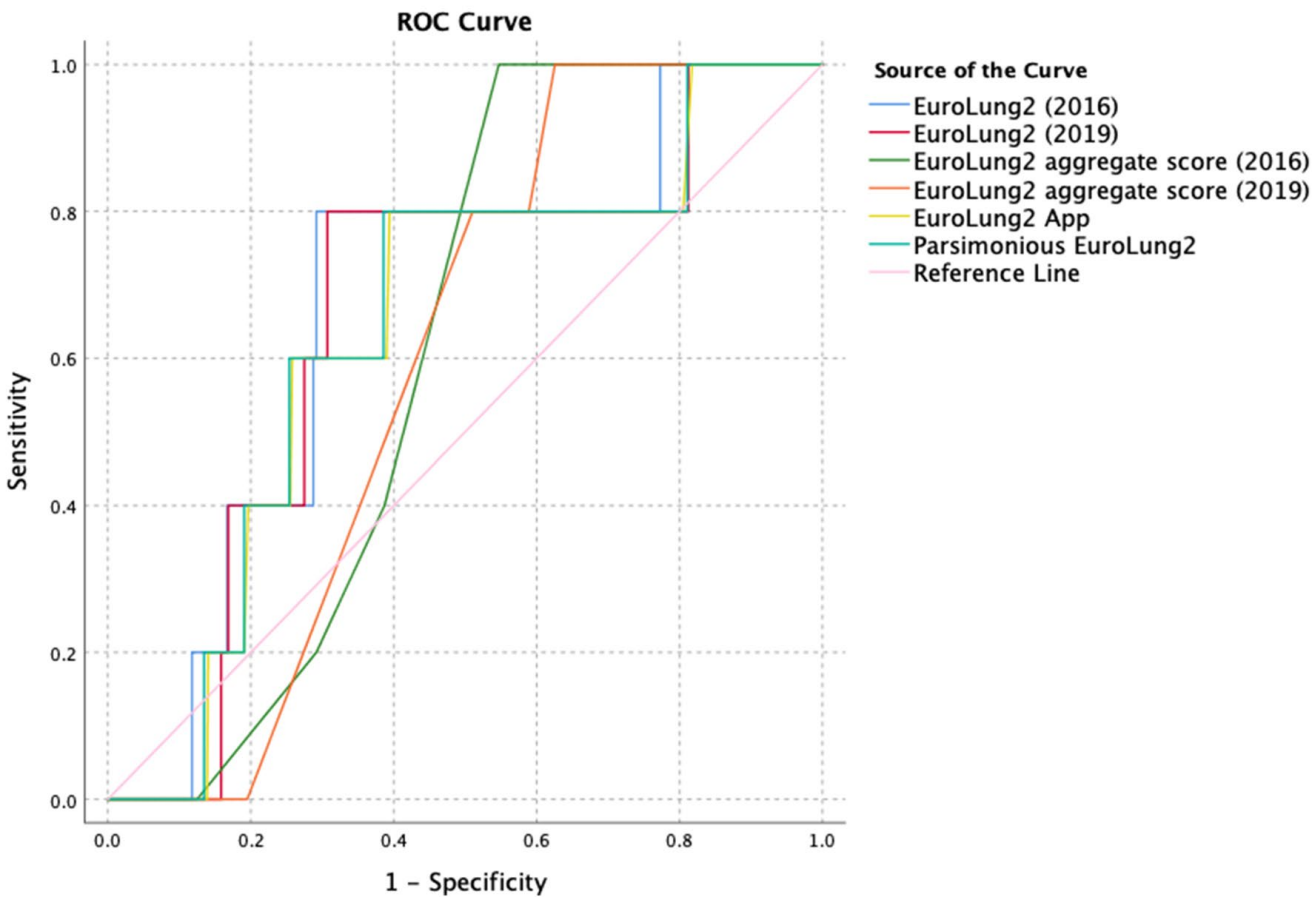
ROC-Curves of EuroLung2 scores

All EuroLung scores showed only a very weak individual correlation (both EuroLung2 aggregate score (2016 & 2019): *η* = 0.025; EuroLung2 (2016): *η* = 0.011; EuroLung2 (2019): *η* = 0.015; parsimonious EuroLung2: *η* = 0.009, EuroLung2 App: *η* = 0.000). In accordance with these results the AUROC was 0.673 for the EuroLung2 (2016), 0.656 for the EuroLung2 (2019), 0.645 for the parsimonious EuroLung2, 0.641 for the EuroLung2 App, 0.610 for the aggregate EuroLung2 (2016) and 0.596 for the aggregate EuroLung2 (2019) and did not proof high discrimination. The AUROC of all available EuroLung scores showed no statistically significant difference, when compared between themselves. Respective ROC curves are shown in Fig. 4.

The Hosmer–Lemeshow test for goodness of fit was not significant for all mortality scores and therefore valid (EuroLung2 (2016 & 2019): *p* = 0.937; parsimonious EuroLung2: *p* = 0.961; EuroLung2 aggregate score (2016): *p* = 0.926; EuroLung2 aggregate score (2019): *p* = 0.313).

For the computational variables, binary logistic regression for the EuroLung2 (2016 & 2019) scores showed lower ppoFEV1% and CAD being significant risk factors for mortality (*p* = 0.040, *p* = 0.033). For the parsimonious EuroLung2 only ppoFEV1% showed significance (*p* = 0.030) and for the EuroLung2 aggregate score (2016) CAD showed significant impact (*p* = 0.025). For the EuroLung2 aggregate score (2019) no significant variable was found.

**Fig. 5:**
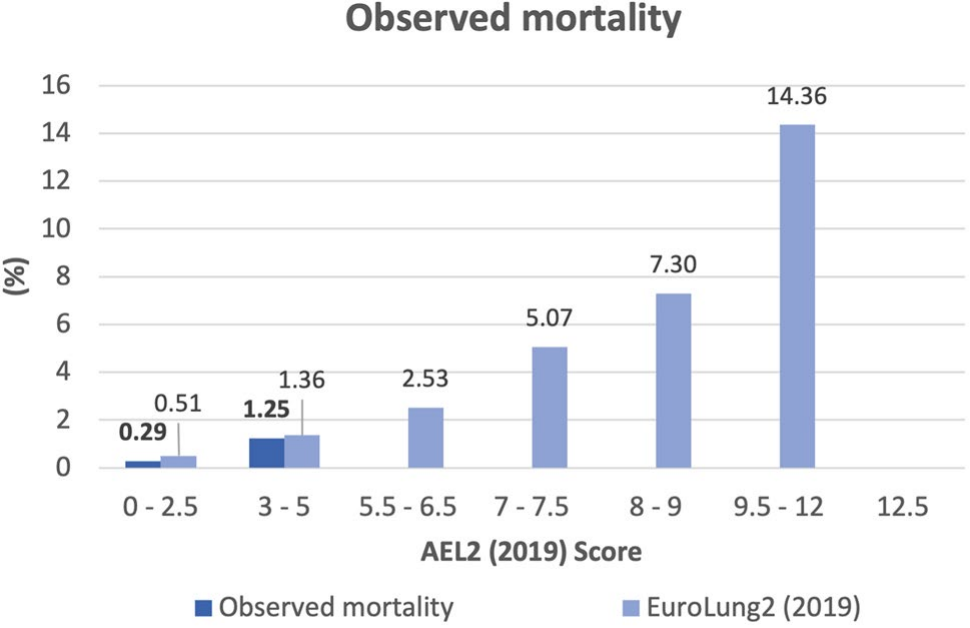
Relationship between EuroLung2 aggregate score and our mortality rates. *AEL2 Score* Aggregate EuroLung2 Score

The relationship of the EuroLung2 aggregate score with our observed mortality rate is shown in Fig. 5.

Two patients died of ARDS, two patients suffered a lethal sepsis and one patient suffered from both complications and died subsequently. Noteworthy, all patients had low EuroLung2 aggregate scores (see Fig. 5). Interestingly, three out of five patients had a history of solid organ transplantation (kidney: *n* = 2, liver: *n* = 1). We found a statistical significant difference in postoperative mortality in the group of patients after solid organ transplantation, compared to the group of non-transplant patients (*p* < 0.001).

A subgroup analysis did not show a difference in observed mortality for patients with neoadjuvant therapy (0 vs. 0.8% in patients without neoadjuvant therapy, *p* = 1.000).

## Discussion

Despite efforts to reduce smoking, lung cancer remains the leading cause of cancer death. To reduce lung cancer associated mortality successful efforts are taken to implement screening routines. As a result more early stage lung cancers are being diagnosed, increasing the number of potentially resectable lung cancers and the demand for individual risk stratification.

The ESTS Eurolung scores were established to calculate individual risk for postoperative morbidity and mortality and to help guiding treatment decisions. So far, the scores have not been definitely validated in other cohorts. The scores can be used in two ways: first, the overall observed morbidity and mortality can be compared to the predicted outcome as a marker for quality of care, comparing a center to the average of the ESTS database; second, the individual predicted risk can be used to guide decision making, but only once the scores have been validated externally.

Aim of this study was to validate the EuroLung scores in our patient cohort, consisting only of primary anatomic VATS resections. As data from our patients are not included in the ESTS database, this could also serve as an external validation.

Our results show that the parsimonious EuroLung1 (2019; 11.11%; 95%CI, 10.74–11.49%) displays the best correlation with our cohort´s observed morbidity rate of 10.45%. Despite this, the correlation with individual patient morbidity was only weak (*η* = 0.155), showing insufficient precision. Although the EuroLung1 (2019) showed a rather good calibration with an intercept of −0.007 and a calibration slope of 0.935 the discrimination was weak with a c-statistics of 0.646.

After performing a binary logistic regression analysis only ppoFEV1% showed to be associated with increased morbidity in our cohort. This emphasizes the importance of preoperative lung function tests in the treatment algorithm of lung cancer. It is even more relevant, as pulmonary prehabilitation programs do show a reduction of postoperative morbidity [12].

Comparing the EuroLung2 scores with our cohort we did show that observed mortality (0.7%) was lower than the one predicted with ESTS EuroLung2 scores. Further analysis showed that lower ppoFEV1% correlated with higher 30-day mortality. Also, we found a high rate of mortality in patients with a history of solid organ transplantation (23.1%). A higher 90-day mortality after surgical treatment of lung cancer in patients after solid organ transplantation was also described recently by Drevet et al. [13]. Solid organ transplantation has so far not been evaluated in the EuroLung Scores, as it is not recorded in the ESTS database, but due to increasing evidence should be considered in future updates.

To investigate possible confounders for this discrepancy between expected and observed morbidity and mortality we compared the patient characteristics of the ESTS database with our own VATS database. Our patients showed a lower ppoFEV1% (72.7 vs. 62.9) and a higher amount of diabetes (2.7% vs. 12.5%). In contrast to the EuroLung database our cohort consists of only VATS patients (vs. 13.1% and 26% in the ESTS database at the time of publication of the EuroLung scores 2016 and 2019), which might decrease postoperative complication rate, as a VATS approach has shown to reduce postoperative morbidity such as pneumonia, intensive care admission, bleeding or the need of reoperation. Even in the case of conversion to open surgery primary VATS cases do not show higher complication rates [4, 14–16]. Analyses of various institutional VATS programs have shown that the surgeon’s experience does not correlate with the amount of major intraoperative complications, but with a higher amount of non-oncological conversions to open surgery during the first 100 cases. This data amplifies the recommendation of Petersen and Hansen for VATS programs and surgeons to be able to perform at least 25 VATS lobectomies per year to complete the respective learning curve in an adequate amount of time and thus hopefully reduce conversion related morbidity [17, 18]. Only a few variables used to calculate EuroLung scores proved to have a significant impact on morbidity and mortality in our cohort.

Regarding postoperative mortality, the lowest predicted number of events was 50% higher than the actual observed mortality (1.1% vs. 0.7%), again showing only weak individual correlation. The reason for the discrepancy is unclear. On the one hand, benefits of minimally invasive surgery might be underestimated in the EuroLung scores due to the low number of VATS procedures in the ESTS database. On the other hand, as shown by Decaluwe et al., almost 25% of 30-day mortality after a scheduled anatomic VATS resection is linked to major intraoperative complications, which cannot be predicted [17]. However, the intraoperative complication rate does not seem to differ between a primary VATS or thoracotomy approach [19, 20]. Moreover, also potential concerns about more extended tumor stages being the reason for higher morbidity rates in thoracotomy can be dismissed as also major pulmonary resections can be safely performed by VATS without an elevated postoperative complication rate [21].

Perhaps future EuroLung scores will perform better on VATS cohorts, as the number of VATS data in the ESTS database is growing. As Moons et al. recommend, a prognostic model not performing well in new populations should rather include the new patient data than establish a new model [22]. Also, we might miss important clinical details that were not covered in the ESTS database, like frailty, sarcopenia, morbid obesity, anemia, solid organ transplantation, or other known risk factors of unfavorable postoperative outcome [13, 23–27].

According to our results, the EuroLung scores can be used to benchmark quality of care in Europe, but should not be used to preclude patients from surgical treatment of lung cancer due to its weak individual correlation. The various risk scores can be used for a more detailed patient consenting, to set expectation within reason, but also to screen for patients who might benefit most from preoperative rehabilitation efforts. The inclusion of other clinical factors such as frailty scores, or sarcopenia screening might improve the accuracy of the risk scores.

## Limitations

The fact that our database consists only of primary VATS patients might influence study outcome, as the prognostic EuroLung scores have been established on a mixed cohort with a rather high thoracotomy rate.

The retrospective character is no limitation of this study as the study design was set as an external model validation study. Although treatment methods and patient selection throughout the years might have changed, it should not impact the validity of our result, because the ESTS database, on which the EuroLung scores are based on, includes patients between June 2007 and December 2018.

Interpretation of our validation of EuroLung2 scores in our study has to be undertaken with caution, as the study population had a rather low number of events. Therefore, also no adequate calibration analysis was possible.

## Conclusion

Decision for or against surgery for lung cancer remains a highly individual decision for each patient and should not be based upon currently available risk scores. A calculated risk score should not inhibit patients from receiving surgery for lung cancer. Risk score calculation should rather be used for improved patient consenting and comparison of postoperative outcome with other departments. Currently, many large retrospective databases, such as the ESTS database, lack promising new risk factors making it difficult if not impossible to establish more precise risk prediction models with these databases. Future efforts should aim at including these variables, such as sarcopenia or history of solid organ transplantation, for further adaptions of the risk score.

## Supplementary Information

Below is the link to the electronic supplementary material.

Supplementary file1 (DOCX 17 kb)

Supplementary file2 (DOCX 17 kb)

Supplementary file3 (DOCX 17 kb)

Supplementary file4 (DOCX 14 kb)
