# Supplementary material for: External validation of risk prediction scores in patients undergoing anatomic video-assisted thoracoscopic resection
Source: Surg Endosc. 2022 Dec 7;37(4):2789–99. doi: 10.1007/s00464-022-09786-7 (PMC10081977; doi:10.1007/s00464-022-09786-7)
Supplement: Supplementary file 1 — Supplementary file1 (DOCX 17 kb) [file 464_2022_9786_MOESM1_ESM.docx]

Supplementary Table 1: Patient characteristics of the study cohorts.

| *Variables* | *Results according to Brunelli et al. (4)* | *Results according to Brunelli et al. (5)* | *Results of the Innsbruck cohort* |
| --- | --- | --- | --- |
| n | 47 960 | 82 383 | 718 |
| Age | 62.6 (11.4) | 64.6 * | 63.6 (10.1) |
| Women | 15 403 (32.1%) | 28 603 (34.7%) | 328 (45.7%) |
| Men | 32 557 (67.9%) | 53 780 (65.3%) | 390 (54.4%) |
| BMI | 25.5 (4.5) | 25.1 * | 25.3 (4.5) |
| ppoFEV_1_% | 72.7 (20.1) | 73 * | 62.9 (15.1) |
| ASA | 2.07 (.7) | n/a | n/a |
| CAD according to ESTS | 3 668 (7.7%) | 6 725 (8.2%) | 62 (8.6%) |
| CVD according to ESTS | 1 303 (2.7%) | 2 434 (3.0%) | 31 (4.3%) |
| CKD | 3 971 (8.3%) | 4 579 (5.6%) | 42 (5.9%) |
| Diabetes | 1 289 (2.7%) | n/a | 90 (12.5%) |
| Neoadjuvant therapy | 4 726 (9.9%) | n/a | 73 (10.2%) |
| Hypertension | n/a | n/a | 292 (40.7%) |
| COPD | n/a | n/a | 232 (32.3%) |
| Hemoglobin (g/dl) (preoperative) | n/a | n/a | 13.78 (3.21) |
| Creatinine mg/dl (preoperative) | n/a | n/a | .93 (.41) |
| VATS | 6 276 (13.1%) | n/a | 718 (100%) |
| Thoracotomy | 41 684 (86.9%) | 61 252 (74.4%) | 0 (0%) |
| Pneumectomy | 5 040 (10.5%) | n/a | 20 (2.8%) |
| Bilobectomy | 2 296 (4.8%) | n/a | 29 (4.0%) |
| Lobectomy | 36 376 (75.8%) | n/a | 618 (86.1%) |
| Segmentectomy | 4 248 (8.9%) | n/a | 34 (4.7%) |
| Extended resections | 2 548 (5.3%) | 4 722 (5.7%) | 10 (1.4%) |
| Observed EuroLung-morbidity | 8 805 (18.4%) | n/a | 75 (10.5%) |
| EuroLung1 (2016) | n/a | n/a | 21.5 (9.3) |
| Observed 30-day-mortality | 1 295 (2.7%) | 1 851 (2.2%) | 5 (.7%) |
| EuroLung2 (2016) | n/a | n/a | 1.43 (1.6) |

Results are shown as mean (standard deviation unless otherwise defined). * Standard Deviation not reported. BMI: body mass index; ppoFEV1: predicted postoperative forced expiratory volume in 1 s; CAD: coronary artery disease; ESTS: European Society of Thoracic Surgeons; CVD: cerebrovascular disease; CKD: chronic kidney disease; COPD: chronic obstructive lung disease; VATS: video-assisted thoracoscopic surgery.
