# Supplementary material for: External validation of risk prediction scores in patients undergoing anatomic video-assisted thoracoscopic resection
Source: Surg Endosc. 2022 Dec 7;37(4):2789–99. doi: 10.1007/s00464-022-09786-7 (PMC10081977; doi:10.1007/s00464-022-09786-7)
Supplement: Supplementary file 2 — Supplementary file2 (DOCX 17 kb) [file 464_2022_9786_MOESM2_ESM.docx]

Supplementary Table 2: Patient characteristics of the morbidity analysis.

| *Variables* | *Observed EuroLung-morbidity in the study by Brunelli et al. (4)* | | | *Observed EuroLung-morbidity in the study by Brunelli et al. (5)* | | | *Observed EuroLung-morbidity in the Innsbruck cohort* | | |
| --- | --- | --- | --- | --- | --- | --- | --- | --- | --- |
|  | *Yes* | *No* | *p* | *Yes* | *No* | *p* | *Yes* | *No* | *p* |
| n | 8 805 | 39 155 |  | 12 955 | 69 428 |  | 75 | 643 |  |
| Age | 65.21 (9.9) | 61.95 (11.6) | <0.001 | n/a | n/a | n/a | 65.61 (9.7) | 63.41 (10.1) | .073 |
| Male sex | 6975 (79%) | 25 582 (65%) | <0.001 | n/a | n/a | n/a | 52 (69.3%) | 338 (52.6%) | .007 |
| BMI | 25.50 (4.7) | 25.49 (4.5) | .9 | n/a | n/a | n/a | 25.30 (5.2) | 25.34 (4.4) | .946 |
| ASA score | 2.28 (.7) | 2.02 (.7) | <0.001 | n/a | n/a | n/a | n/a | | |
| ppoFEV_1_% | 67.90 (23.1) | 73.81 (19.2) | <0.001 | n/a | n/a | n/a | 58.61 (14.2) | 63.43 (15.1) | .009 |
| CAD according to ESTS | 932 (10.6%) | 2 736 (7.0%) | <0.001 | n/a | n/a | n/a | 9 (12.0%) | 53 (8.2%) | .277 |
| CVD according to ESTS | 350 (4.0%) | 9 53 (2.4%) | <0.001 | n/a | n/a | n/a | 6 (8.0%) | 25 (3.9%) | .124 |
| CKD | 812 (9.2%) | 3 159 (8.1%) | <0.001 | n/a | n/a | n/a | 9 (12.0%) | 33 (5.1%) | .032 |
| Diabetes | 281 (3.2%) | 1 008 (2.6%) | .001 | n/a | n/a | n/a | 15 (20.0%) | 75 (11.7%) | .044 |
| Neoadjuvant therapy | 956 (10.9%) | 3 770 (9.6%) | <0.001 | n/a | n/a | n/a | 9 (12.0%) | 64 (10.0%) | .686 |
| Thoracotomy* | 8 083 (91.8%) | 33 601 (85.8%) | <0.001 | n/a | n/a | n/a | 0 (0%) | 0 (0%) |  |
| Extended resection | 746 (8.5%) | 1 802 (4.6%) | <0.001 | n/a | n/a | n/a | 3 (4.0%) | 7 (1.1%) | .077 |
| Pneumectomy | n/a | n/a | n/a | n/a | n/a | n/a | 0 (0%) | 20 (3.1%) | .253 |
| Hemoglobin (g/dl) (preoperative) | n/a | n/a | n/a | n/a | n/a | n/a | 14.65 (9.1) | 13.68 (1.4) | .014 |
| Creatinine mg/dl (preoperative) | n/a | n/a | n/a | n/a | n/a | n/a | .99 (.4) | .93 (.4) | .255 |

Results are shown as mean (standard deviation unless otherwise defined). *Thoracotomies and conversions are combined, since no detailed information is available in this regard. BMI: body mass index; ASA: American Society of Anesthesiologists; ppoFEV1: predicted postoperative forced expiratory volume in 1 s; CAD: coronary artery disease; ESTS: European Society of Thoracic Surgeons; CVD: cerebrovascular disease; CKD: chronic kidney disease.
