# Supplementary material for: External validation of risk prediction scores in patients undergoing anatomic video-assisted thoracoscopic resection
Source: Surg Endosc. 2022 Dec 7;37(4):2789–99. doi: 10.1007/s00464-022-09786-7 (PMC10081977; doi:10.1007/s00464-022-09786-7)
Supplement: Supplementary file 3 — Supplementary file3 (DOCX 17 kb) [file 464_2022_9786_MOESM3_ESM.docx]

Supplementary Table 3: Patient characteristics of the mortality analysis.

| *Variables* | *Observed EuroLung-mortality in the study by Brunelli et al. (4)* | | | *Observed EuroLung-mortality in the study by Brunelli et al. (5)* | | | *Observed EuroLung-mortality in the Innsbruck cohort* | | |
| --- | --- | --- | --- | --- | --- | --- | --- | --- | --- |
|  | *Yes* | *No* | *p* | *Yes* | *No* | *p* | *Yes* | *No* | *p* |
| n | 1 295 | 46 665 |  | 1 851 | 80 532 |  | 5 | 713 |  |
| Age | 67.08 (10.0) | 62.43 (11.4) | <0.001 | n/a | n/a | n/a | 65.40 (9.9) | 63.63 (10.1) | .865 |
| Male sex | 1120 (86.5%) | 31 437 (67.4%) | <0.001 | n/a | n/a | n/a | 3 (60.0%) | 390 (54.7%) | 1.000 |
| BMI | 24.79 (4.5) | 25.51 (4.5) | <0.001 | n/a | n/a | n/a | 22.13 (2.9) | 25.36 (4.5) | .111 |
| ASA score | 2.49 (.7) | 2.06 (.7) | <0.001 | n/a | n/a | n/a | n/a | | |
| ppoFEV_1_% | 66.39 (25.6) | 72.91 (19.9) | <0.001 | n/a | n/a | n/a | 47.89 (8.0) | 63.03 (15.1) | .025 |
| CAD according to ESTS | 152 (11.7%) | 3 516 (7.5%) | <0.001 | n/a | n/a | n/a | 2 (40.0%) | 60 (8.4%) | .062 |
| CVD according to ESTS | 70 (5.4%) | 1 233 (2.6%) | <0.001 | n/a | n/a | n/a | 0 (0%) | 31 (4.4%) | 1.000 |
| CKD | 104 (8.0%) | 3 867 (8.3%) | .7 | n/a | n/a | n/a | 2 (40.0%) | 40 (5.6%) | .030 |
| Diabetes | 46 (3.6%) | 1 243 (2.7%) | .05 | n/a | n/a | n/a | 1 (16.7%) | 89 (12.5%) | .489 |
| Neoadjuvant therapy | 129 (10.0%) | 4 597 (9.9%) | .9 | n/a | n/a | n/a | 0 (0%) | 73 (10.2%) | 1.000 |
| Thoracotomy* | 1 237 (95.5%) | 40 447 (86.7%) | <0.001 | n/a | n/a | n/a | 0 (0%) | 0 (0%) |  |
| Extended resection | 134 (10.3%) | 2 414 (5.2%) | <0.001 | n/a | n/a | n/a | 0 (0%) | 10 (1.4%) | 1.000 |
| Pneumectomy | 345 (26.6%) | 4 695 (10.1%) | <0.001 | n/a | n/a | n/a | 0 (0%) | 20 (2.8%) | 1.000 |
| Hemoglobin (g/dl) (preoperative) | n/a | n/a | n/a | n/a | n/a | n/a | 12.6 (1.1) | 13.8 (3.2) | .443 |
| Creatinine mg/dl (preoperative) | n/a | n/a | n/a | n/a | n/a | n/a | 1.56 (.8) | .93 (.4) | .001 |

Results are shown as mean (standard deviation unless otherwise defined). *Thoracotomies and conversions are combined, since no detailed information is available in this regard. BMI: body mass index; ASA: American Society of Anesthesiologists; ppoFEV1: predicted postoperative forced expiratory volume in 1 s; CAD: coronary artery disease; ESTS: European Society of Thoracic Surgeons; CVD: cerebrovascular disease; CKD: chronic kidney disease.
