# Supplementary material for: External validation of risk prediction scores in patients undergoing anatomic video-assisted thoracoscopic resection
Source: Surg Endosc. 2022 Dec 7;37(4):2789–99. doi: 10.1007/s00464-022-09786-7 (PMC10081977; doi:10.1007/s00464-022-09786-7)
Supplement: Supplementary file 4 — Supplementary file4 (DOCX 14 kb) [file 464_2022_9786_MOESM4_ESM.docx]

Supplementary Table 4: Postoperative complications.

| *Type of postoperative complication* |  | *n** | *%* | |
| --- | --- | --- | --- | --- |
| Respiratory failure | | 5 | | .70 |
| Prolonged mechanical ventilation >24h | | 2 | | .28 |
| Acute cardiac failure | | 1 | | .14 |
| Need for reintubation | | 4 | | .56 |
| Pneumonia | | 24 | | 3.34 |
| Atelectasis requiring bronchoscopy | | 23 | | 3.20 |
| Pulmonary oedema | | 0 | | 0 |
| Pulmonary embolism | | 0 | | 0 |
| ARDS/acute lung injury | | 1 | | .14 |
| Arrythmia requiring treatment | | 22 | | 3.06 |
| Acute myocardial ischemia | | 1 | | .14 |
| Stroke/transient ischemic attacks | | 0 | | 0 |
| Acute kidney injury | | 1 | | .14 |

ARDS: Acute Respiratory Distress Syndrome. *some patients suffered from more than one complication
